# Synergistic effects of combined platelet-activating factor receptor and epidermal growth factor receptor targeting in ovarian cancer cells

**DOI:** 10.1186/1756-8722-7-39

**Published:** 2014-05-06

**Authors:** Yi Yu, Mingxing Zhang, Xiaoyan Zhang, Qingqing Cai, Shanshan Hong, Wei Jiang, Congjian Xu

**Affiliations:** 1 Obstetrics and Gynecology Hospital, Fudan University, No.419 Fang-Xie Road, Shanghai, 200011, People’s Republic of China; 2 Department of Obstetrics and Gynecology of Shanghai Medical School, Fudan University, No.138 Yi-Xueyuan Road, Shanghai 200032, People’s Republic of China; 3 Shanghai Key Laboratory of Female Reproductive Endocrine Related Diseases, No. 413 Zhao-Zhou Road, Shanghai 200011, People’s Republic of China; 4 Institute of Biomedical Sciences, Fudan University, Shanghai 200032, No.138 Yi-Xueyuan Road, Shanghai 200032, People’s Republic of China

**Keywords:** Platelet-activating factor receptor (PAFR), Epidermal growth factor receptor (EGFR), Ovarian cancer, Combined-targeting, Signal pathway

## Abstract

**Background:**

Genetic alterations, including the overexpression of epidermal growth factor receptor (EGFR), play a crucial role in ovarian carcinogenesis. To date, EGFR targeting has shown limited antitumor effects in ovarian cancer when administered as monotherapy. We previously identified platelet-activating factor receptor (PAFR) as being overexpressed in ovarian cancer and found that its ligand PAF evoked EGFR phosphorylation. To determine whether PAFR targeting can enhance the antitumor efficacy of EGFR inhibition, we investigated the effects of a PAFR antagonist (WEB2086) in conjunction with an EGFR inhibitor (AG1478).

**Methods:**

The expression of EGFR and PAFR in CAOV-3 and SKOV-3 ovarian cancer cell lines was measured by Western blot and immunocytochemistry. Synergy was determined using isobologram analysis. The effects of combined PAFR and EGFR targeting on both cells were assessed by using CCK-8, transwell, flow cytometry, western blot analysis. In vivo studies were conducted using CAOV-3 cells xenografted in nu/nu mice.

**Results:**

Treatment with combination WEB2086 and AG1478 resulted in significantly greater inhibition of proliferation and invasion compared to either drug alone. When examining equipotent combinations of WEB2086 and AG1478 to determine potential synergy, a combination index (CI) of 0.49 was identified for CAOV-3 cells and a CI of 0.58 for SKOV-3 cells indicating synergy. This co-inhibition induced significantly more apoptosis and arrested the cells at G0/G1 phase in both cell lines. The activation of PAFR and/or EGFR induced phosphorylation of the mTOR, AKT, and MAPK pathways. Combined PAFR and EGFR targeting synergistically diminished the expression of PAFR and EGFR phosphorylation and downstream signaling. In vivo studies further verified the antitumor effects of combined PAFR and EGFR targeting in a CAOV-3 xenograft model.

**Conclusions:**

These results suggest that WEB2086 and AG1478 are synergistic in ovarian cancer cells with high expression of both PAFR and EGFR. The presented approach may have important therapeutic implications in the treatment of ovarian cancer patients.

## Background

Ovarian cancer is the fifth most common cause of death from all cancers among women in the world and has the highest mortality rate of gynecological cancers [1]. Overall, ovarian cancer has the worst prognosis of all gynecological cancers, with a 5-year survival rate of less than 40% [2]. Surgical resection and platinum-based combination regimens offer a modest but significant survival advantage in ovarian cancer patients with advanced or metastatic disease, though most patients eventually experience disease progression. Advances in the understanding of the molecular biology of cancer have enabled the discovery of several potential molecular targets and the development of novel targeted therapies.

Epidermal growth factor receptor (EGFR) is involved in the development and progression of several human cancers, including ovarian cancer. The most common type of ovarian cancer arises from ovarian surface epithelium, tissue that commonly expresses EGFR [3]. Approximately 70% of ovarian tumors express activated EGFR [4]. EGFR is a transmembrane receptor that plays a significant role in neural development and the formation of skin. EGFR also plays a role in various pro-survival and anti-apoptotic pathways in cancer cells [5-7]. Furthermore, EGFR is also involved in cell migration, metastasis, angiogenesis, and the epithelial mesenchymal transition (EMT) [8-10]. However, recent clinical trials targeting EGFR with cetuximab [11-13], matuzumab [14,15], gefitinib [16], and erlotinib [17,18] in epithelial ovarian cancer patients have shown only modest clinical responsiveness. The modest responses of EGFR blockade when monoclonal antibodies or tyrosine kinase inhibitors are administered as single agents could be attributed to compensation by other signaling pathways [19].

Various ligands such as epidermal growth factor (EGF) and transforming growth factor (TGF) can activate EGFR. Our previous studies have demonstrated that platelet-activating factor (PAF) also induced increased EGFR phosphorylation [20]. PAF is one of major phospholipid mediators functioning in many different biological pathways in inflammatory diseases and cancers. PAF induces diverse biological effects through its specific receptor, PAFR, which belongs to the G-protein coupled receptor (GPCR) family [21-23]. We have demonstrated that the PAFR gene and protein are overexpressed in ovarian cancer tissues and cells and that PAF can promote the proliferation and invasion of ovarian cancer cells in a PAFR-dependent manner. These results suggest that activated EGFR and PAFR may synergistically promote the progression of ovarian cancer and that the constitutive activation of EGFR and downstream signaling pathways by PAFR may contribute to the inefficacy of EGFR inhibitors in ovarian cancer.

The aim of the present work was to determine whether the addition of PAFR targeting can enhance the antitumor efficacy of EGFR tyrosine kinase inhibitors. The PAFR antagonist WEB2086 was combined with the EGFR inhibitor AG1478 in ovarian cancer in vitro and in vivo. The effects of the two agents, alone and in combination, were determined in vitro and in vivo and the underlying molecular mechanisms were assessed.

## Materials and methods

### Cell culture and chemical reagents

The ovarian cancer cell lines CAOV-3 and SKOV-3 (purchased from the Cell Bank of the Chinese Academy of Science, Shanghai, China) were cultured at 37°C in a humidified 5% CO_2_ atmosphere in RPMI-1640 medium with 10% fetal calf serum (Gibco, Invitrogen, Carlsbad, CA), 100 IU/ml penicillin G, and 100 mg/ml streptomycin sulfate (Sigma-Aldrich, St. Louis, MO). AG1478 (EGFR inhibitor) [24] and WEB2086 (PAFR antagonist) [25] were purchased from Sigma-Aldrich (St Louis, MO). A Cell Counting Kit 8 (CCK8) was purchased from Dojindo Molecular Technologies, Inc. (Kumamoto, Japan), and the Alexa Fluor 488 annexin V/Dead Cell Apoptosis Kit was obtained Invitrogen (Carlsbad, CA). Rabbit polyclonal antibodies directed against PAFR, cleaved-caspase3, cleaved-PARP, phospho/total- EGFR, phospho/total- β-arrestin2, phospho/total- P70S6K, phospho/total- AKT, phospho/total- 4EBP1, and phospho/total- ERK were used in this study. All of these antibodies were purchased from Cell Signaling Technology Co. Mouse monoclonal antibodies directed against actin were also used (Sigma, Missouri, USA).

### Western blot analysis

Cellular extracts were prepared in modified radioimmunoprecipitation assay (RIPA) buffer (50 mM Tris–HCl pH 7.4, 1% NP-40, 0.25% Na-deoxycholate, 150 mM NaCl, 1 mM EDTA, 1 mM PMSF, and protease inhibitor cocktail). The protein concentrations of the cellular extracts were measured using a Bio-Rad protein assay kit. The cellular extracts were subjected to SDS-PAGE, and the proteins were transferred to PVDF membranes. After blocking for 1 h at room temperature in 5% BSA, the blots were probed with the primary antibody at a 1:1000 dilution and incubated overnight at 4°C. Subsequently, the blots were washed three times and incubated for 1 h at room temperature with a 1:10000 dilution of the secondary peroxidase-conjugated antibodies. Following three washes, the immunoreactive bands were detected using electrochemiluminescence (ECL).

### Immunocytochemistry

Immunocytochemistry was used to detect the expression of EGFR and PAFR. After fixation with 4% paraformaldehyde, cells were incubated with an anti-EGFR (1:50) or -PAFR (1:50) antibody overnight, and then incubated with peroxidase-conjugated anti-rabbit IgG for 30 min. The staining reaction was performed with diaminobenzidine. The cells were counter-stained with hematoxylin to detect nuclei, and imaged using light microscopy (Olympus, Tokyo, Japan).

### Cell proliferation and invasion assay

Cells were seeded in 96-well plates and treated with different doses of WEB2086, AG1478, or both for 72 hours. Cell proliferation was measured with the CCK-8 assay. A 10-μL aliquot of CCK-8 solution was added to 100 μL medium in each well for 1–4 hours, and the absorbance was measured at 450 nm. The percentage of cell viability was determined relative to the control. Each experiment was performed in six replicate wells for each drug concentration. The IC50 values were calculated with the SPSS software using the bliss method.

Cell invasion activity assay were conducted with an 8 μm matrigel invasion chamber (Corning, NY). Experiments were carried out according to the manufacture’s protocal. Briefly, each well insert was coated with 100 μl mixture matrigel: serum-free medium, followed by incubation at 37°C for 4 hours. Cells were then trypsin digested and transferred into top matrigel wells (10^5^ cancer cells per well) with WEB2086 (0.1 mM), AG1478 (10 μM) or both. 600 μl of 10% FBS media was added to the bottom of the chamber and incubated for 48 hours in the invasion assay. Invasion activity was assessed by the number of cells that crossed the matrigel and filter membrane. Cell numbers were counted under a light microscope and presented as percentage compared to the controls. Experiments were performed twice, at least three repeats for each treatment.

### Assessment of apoptosis and cell cycle

Apoptosis was detected by flow cytometry via the examination of altered plasma membrane phospholipid packing by the lipophilic dye Annexin V as described elsewhere. Briefly, cells were treated with inhibitors and harvested at 24 hours after treatment. The cells were washed twice with PBS and then resuspended in binding buffer at a concentration of 1 × 10^6^ cells/mL according to the manufacturer’s instructions. Thereafter, 5 μL of Annexin V-FITC and 1 μL of propidium iodide were added to 100 μL of cell suspension and incubated for 20 min at room temperature in the dark. After adding 400 μL of binding buffer, the labeled cells were counted by flow cytometry within 1 hour. All early apoptotic cells (Annexin V-positive, propidium iodide -negative), necrotic/late apoptotic cells (double positive), and living cells (double negative) were detected using a FACSCalibur flow cytometer and subsequently analyzed by Cell Quest software. For the cell cycle analyses, treated cells were fixed in 70% ethanol and stored at -20°C overnight; the cells were labeled with propidium iodide (50 μg/ml) and RNase (100 μg/ml) for 30 min before flow cytometry analysis.

### Animals and treatments

Female athymic nu/nu mice (4 to 6 weeks old) were obtained from the Laboratory Animal Center of the Shanghai Institutes For Biological Sciences of the Chinese Academy of Sciences. All animal studies were conducted in strict accordance with protocols approved by the Ethics Committee for Animal Experimentation of Fudan University. A total of 2 × 10^6^ CAOV-3 cells was injected into the flanks of Female athymic nu/nu mice. When established tumors of approximately 75 mm^3^ in Diameter were detected, the mice were randomly divided into four groups (8 mice/group), and subjected to various treatments. The PAFR antagonist WEB2086 was intraperitoneally injected at 5 mg/kg every three days for 2 weeks, and the EGFR inhibitor AG1478 was intraperitoneally injected at 10 mg/kg every three days for 2 weeks. After 3 weeks, all of the mice were killed, and the tumors were excised and weighed. The tumor size for the xenografts was determined using a caliper, and the volume was calculated as = length × width^2^/2, where the width is the smallest measurement and the length is the longest measurement.

### Isobologram analysis

Synergy between WEB2086 and AG1478 was determined by isobologram analysis using CalcuSyn software (Biosoft, Cambridge, UK), which analyzes the data from proliferation assays to determine the interaction between equipotent drug combinations [26]. The combination index values <1, =1 and >1 indicate synergy, additivity and antagonism, respectively.

### Statistical analysis

All experiments were performed at least three times. The data are expressed as the “mean ± SD”. Wherever appropriate, the data were also subjected to unpaired two-tailed Student’s t-tests. Differences were considered significant when *P* < 0.05.

## Results

### Synergistic interaction between the PAFR antagonist WEB2086 and the EGFR inhibitor AG1478

**Figure 1:**
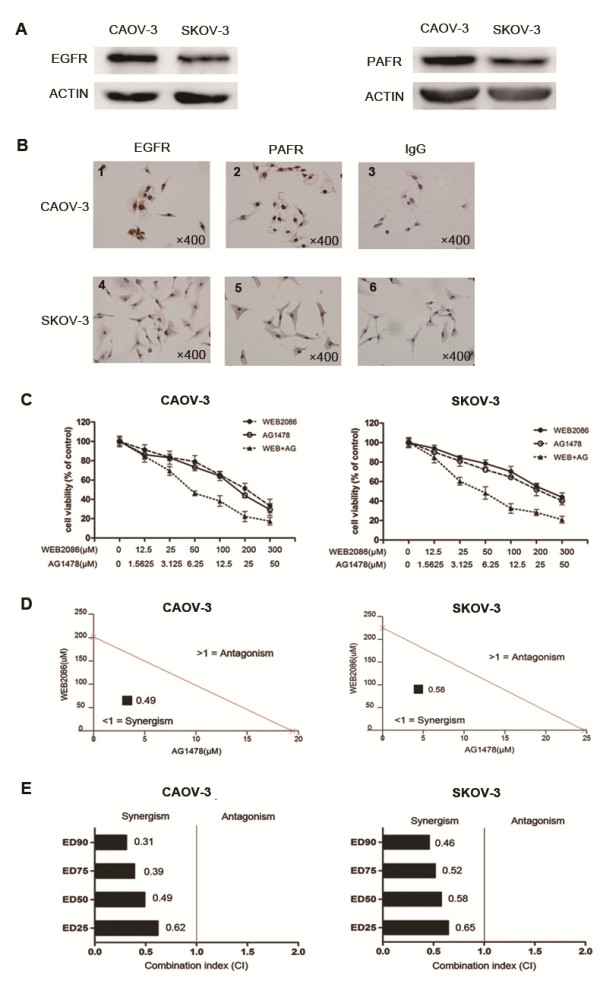
**WEB2086 and AG1478 exhibit synergistic cytotoxicity in CAOV-3 and SKOV-3 cells. (A)** Under basal growth conditions, whole-cell extracts obtained from CAOV-3 and SKOV-3 cells were analyzed for EGFR and PAFR. **(B)** Detection of EGFR and PAFR expression by immunocytochemistry. 1, 2, 3 for CAOV-3 cells and 4, 5, 6 for SKOV-3 cells stained with anti-EGFR antibody, -PAFR antibody, and control IgG, respectively. **(C)** Cellular viability was measured by the CCK-8 assay. CAOV-3 and SKOV-3 cells were treated with the indicated concentrations of AG1478 and WEB2086 for 72 hours. **(D)** Isobologram analysis of combination WEB2086 with AG1478 used in equipotent concentrations in CAOV-3 and SKOV-3 cells. The line designates the CI where CI = 1 (additive effect). CI < 1 indicates synergism and CI > 1 represents antagonism. The combination data points (CI = 0.49 for CAOV-3 and CI = 0.58 for SKOV-3) calculated by CalcuSyn softwre indicate synergism at ED50. **(E)** The CI values of combination WEB2086 and AG1478 at a range of EDs. The CI at ED25, ED50, ED75 and ED90 indicate a synergistic interaction between WEB2086 and AG1478 in CAOV-3 and SKOV-3 cells.

As we previously reported that inhibition of PAFR decrased ovarian cancer cell proliferation and the EGFR inhibition has shown promise in clinical trials in various types of cancers, including ovarian cancer, we asked whether simultaneously targeting the two receptors would have synergistic effects. The inhibitory effects of combined use of the PAFR antagonist WEB2086 and the EGFR inhibitor AG1478 were tested in two ovarian cancer cell lines, CAOV-3 and SKOV-3 cells. We first examined the basal levels of PAFR and EGFR in both cells. As shown in Figure 1A and B, both cells express PAFR and EGFR, while the expression levels of PAFR and EGFR were higher in CAOV-3 cells compared with that in SKOV-3 cells. To determine whether WEB2086 and AG1478 exhibit a combined effect in ovarian cancer cells, we examined the effect of individual and combination treatment with WEB2086 and AG1478 after 72-h exposure using the CCK-8 assay. To ensure that the contributing effects from each drug was equivalent, IC50 for each was determined and serial dilutions were generated based on the IC50 of each drug providing 1:1 equipotent WEB2086/AG1478 ratio. We found that both compounds inhibited cell growth in a dose-dependent manner in CAOV-3 and SKOV-3 cells (Figure 1C). To determine whether WEB2086 and AG1478 interact synergistically, isobologram analysis was performed. This analysis provides a CI value that measures the degree of interaction between two or mor drug, where a CI < 1 and a CI > 1 indicates synergism and antagonism, respectively. A CI of 0.49 and 0.58 was indentified for CAOV-3 and SKOV-3 cells, when the effective dose (ED) of both agents inhibited cell viability by 50% (Figure 1D). As shown in Figure 1E, inrespective of high cytotoxicity (ED90) or low cytotoxicity (ED25), the CI values was remained below 1, indicating that synergism occurs independently of the equipotency levels of WEB2086 and AG1478. Our results demonstrate that WEB2086 and AG1478 exhibit a synergistic interaction in ovarian cancer cells, particularly in CAOV-3 cells.

### Combined targeting of PAFR and EGFR inhibits ovarian cancer cell growth and invasion

**Figure 2:**
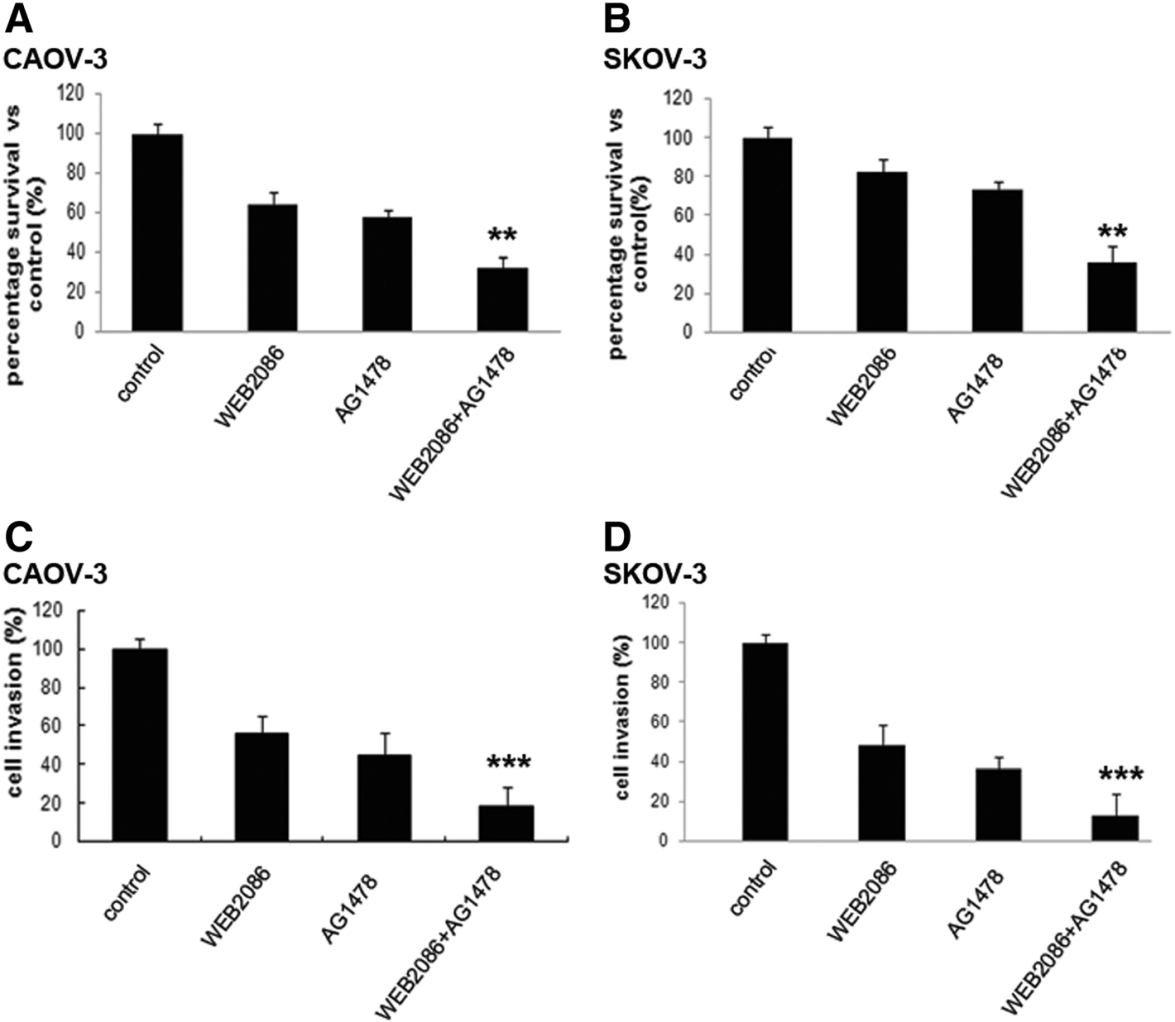
**Combination of PAFR and EGFR targeting decreases cell growth and invasion in CAOV-3 and SKOV-3 cell lines.** Cells were treated with WEB2086 (0.1 mM), AG1478 (10 μM), and their combination. **(A and B)** The percentage of cell survival was measured by the CCK-8 assay 72 hours later. Independent experiments were repeated three times. Columns, means of 3 identical wells of a single representative experiment; bars, upper 95% confidence interval; **, *p* < 0.005 for comparisons between cells treated with the combined treatment and cells treated with the single agent. **(C and D)** Cells were plated in Matrigel invasion chambers and invading cells were counted using light microscopy. Columns, means of 3 identical wells of a single representative experiment; bars, upper 95% confidence interval; ***, *p* < 0.001 for comparisons between cells treated with the combined treatment and cells treated with the single agent.

To test whether the combined targeting of PAFR and EGFR resulted in enhanced growth inhibition compared with the single inhibition, half of the IC50 for each drug was used to treat ovarian cancer cells, followed by the CCK-8 assay. As shown in Figure 2A and B, the combined inhibition of both PAFR and EGFR resulted in significantly enhanced growth inhibition compared with either treatment alone over a 72-hour exposure. The results in CAOV-3 and SKOV-3 cell lines confirmed a strong inhibitory effect by adding WEB2086 to AG1478.

We previously reported that PAF promots ovarian cancer cell invasion. In addition to cell growth, we examined the effects on cell invasion of combined PAFR and EGFR targeting in ovarian cancer cells. As shown in Figure 2C and D, although WEB2086 or AG1478 alone decreased ovarian cancer cell invasion, combined targeting significantly enhanced the effect when compared with either treatment alone.

### Combined targeting of PAFR and EGFR increases ovarian cancer cell apoptosis and G0/G1 arrest

**Figure 3:**
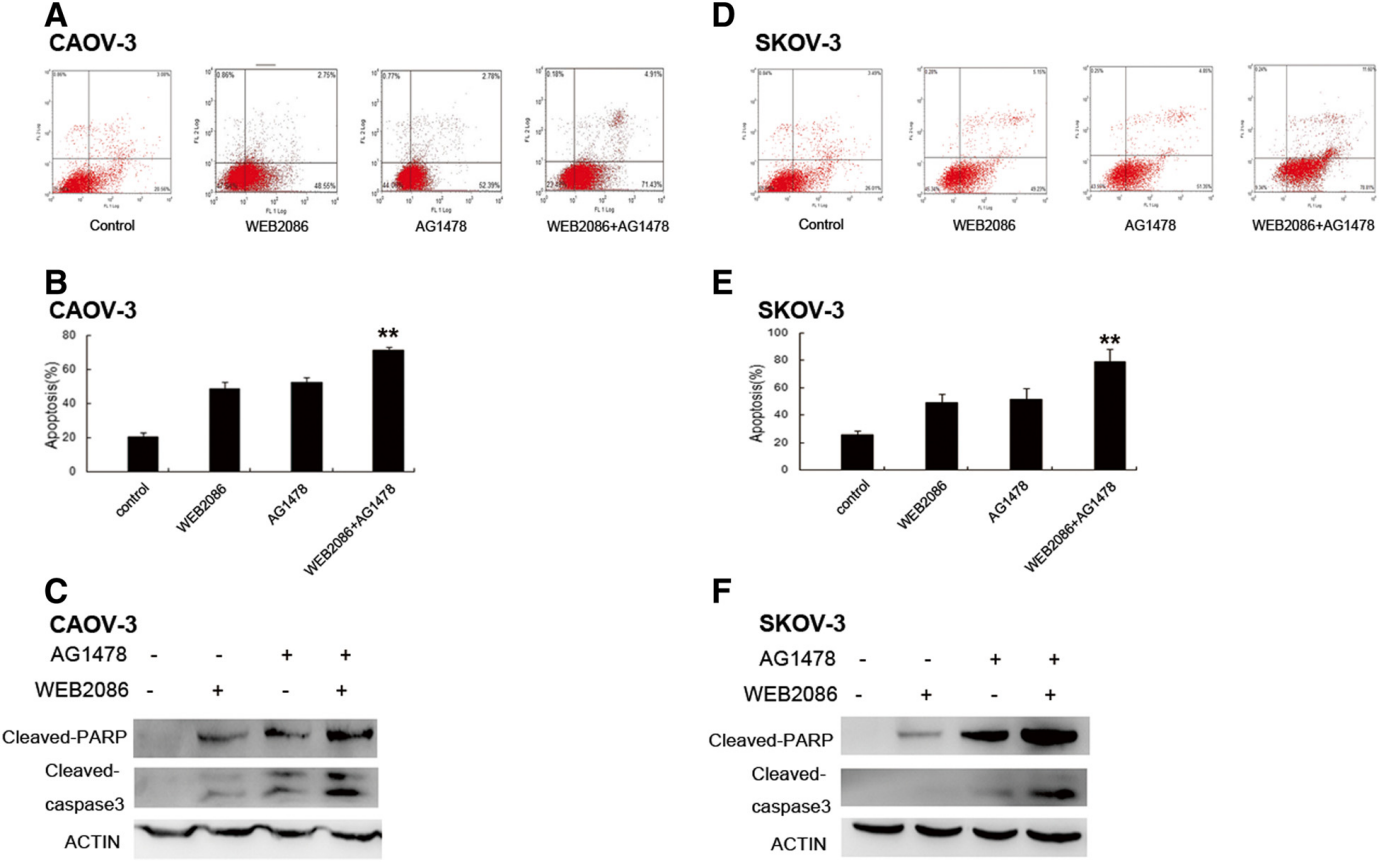
**Effects of WEB2086 and/or AG1478 on apoptosis in CAOV-3 and SKOV-3 cell lines.** Apoptosis was evaluated as described in the Materials and Methods with Annexin V staining in CAOV-3 and SKOV-3 cells; the cells were treated with WEB2086 (0.1 mM), AG1478 (10 μM), and their combination. **A and D**: Representative dot plots illustrating the data near the mean of the groups in **B and E**. Columns, means of 3 identical wells of a single representative experiment; bars, upper 95% confidence interval; **, *p* < 0.005 for comparisons between cells treated with the combined treatment and cells treated with the single agent. **C and F**: Western blotting analysis of PARP and caspase-3 cleavage following treatment with WEB2086 alone or with AG1478. Western blotting of β-Actin is included as a loading control.

**Figure 4:**
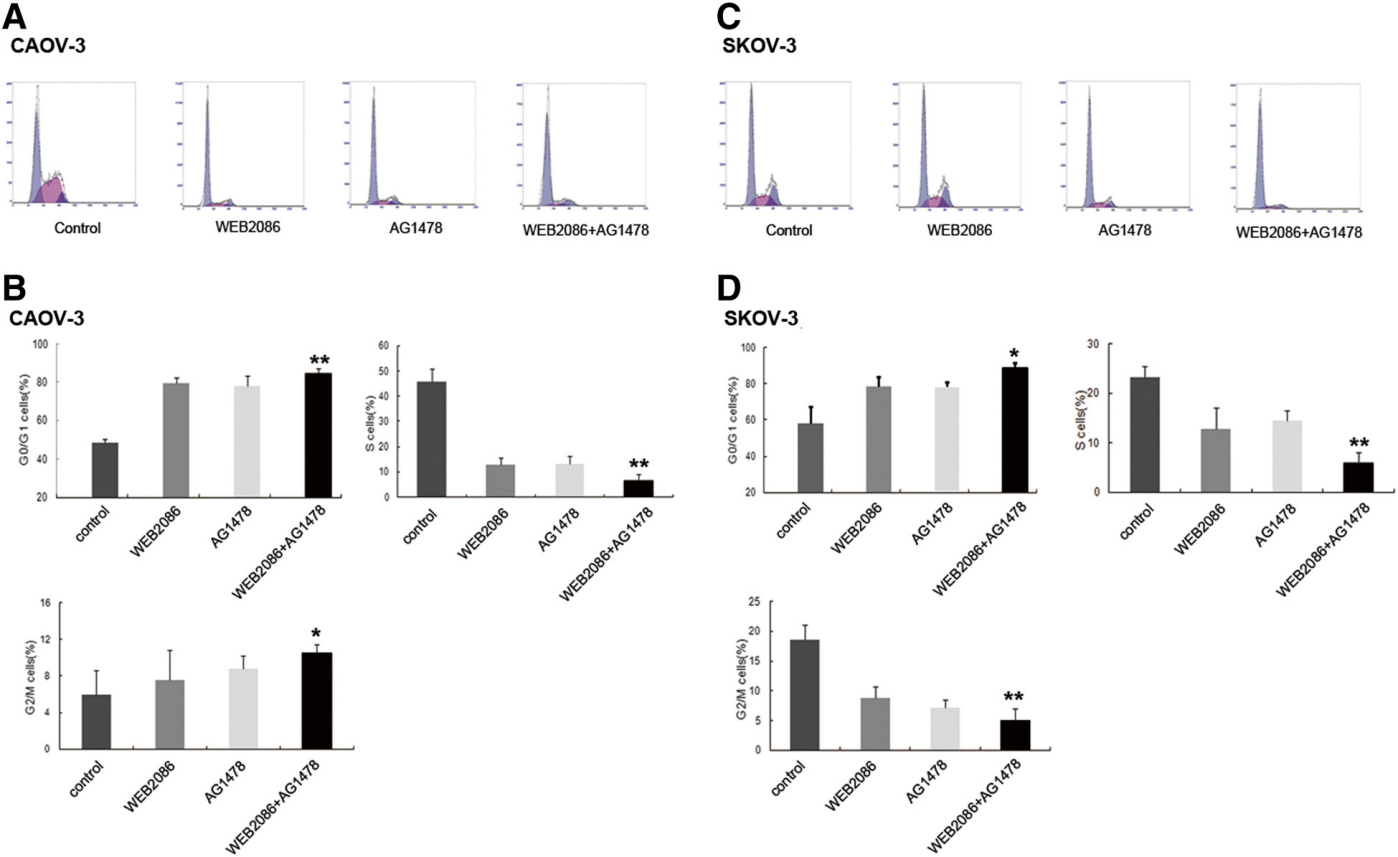
**Effect of WEB2086 and/or AG1478 on the cell cycle in CAOV-3 and SKOV-3 cell lines.** The cell cycle was assessed as described in the Materials and Methods with propidium iodide and RNase staining of CAOV-3 and SKOV-3 cells; the cells were treated with WEB2086 (0.1 mM), AG1478 (10 μM), and their combination. **A and C**: Representative dot plots illustrating the data near the mean of the groups in **B and D**. Columns, means of 3 identical wells of a single representative experiment; bars, upper 95% confidence interval; *, *p* < 0.05 and **, *p* < 0.005 for comparisons between cells treated with the combined treatment and cells treated with the single agent.

To determine whether the increased antiproliferative effect was due to increased apoptosis and/or cell cycle alterations, we examined apoptosis by Annexin V analysis following WEB2086 and AG1478 treatment. The number of apoptotic cells was quantified (Figure 3A and D). As shown in Figure 3B and E, a flow cytometric analysis of CAOV-3 and SKOV-3 cells revealed that both WEB2086 and AG1478 increased the number of apoptotic cells compared to that observed in the untreated cells; additionally, the combined targeting significantly enhanced CAOV-3 and SKOV-3 cell apoptosis to 71.43% and 78.81%, respectively. These results were confirmed by a western blot analysis. Both WEB2086 and AG1478 were able to induce the cleavage of 113-kDa poly-ADP-ribose polymerase (PARP) to the 89-kDa fragment and induce cleaved caspase-3 in CAOV-3 and SKOV-3 cells, and the combination of WEB2086 and AG1478 was accompanied by increased expression of cleaved PARP and caspase-3 (Figure 3C and F). As shown in Figure 4, a significant increase in G0/G1-phase cells after treatment with WEB2086 combined with AG1478 when compared with the single treatment in CAOV-3 and SKOV-3 cells was observed. This cell cycle delay was also accompanied by a decreased percentage of S-phase cells.

### Identification of intracellular signaling pathways following the activation of PAFR and EGFR

**Figure 5:**
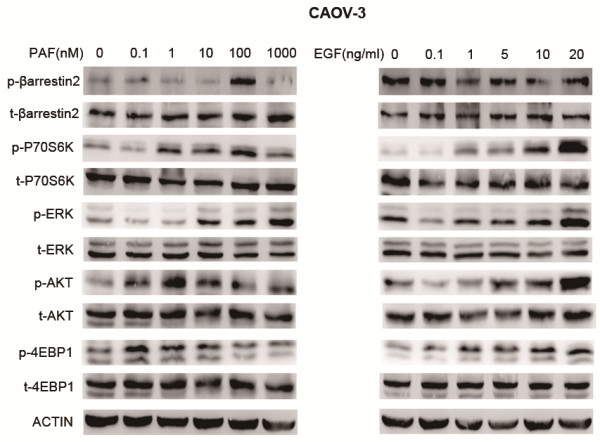
**Effects on downstream pathways following the activation of PAFR and EGFR in CAOV-3 cells.** CAOV-3 cell lysates were subjected to western blotting of phospho/total- β-arrestin2, phospho/total- P70S6K, phospho/total- ERK, phospho/total- AKT, and phospho/total- 4EBP1 activation following treatment with the indicated concentrations of PAF and EGF for 24 hours. β-Actin was included as a loading control.

Enhanced antitumor effects were observed by combined PAFR and EGFR targeting, which indicates that intracellular signaling pathway crosstalk may occur between the activated PAFR and EGFR pathways. We next evaluated the effects of activated PAFR and EGFR on the expression of selected proteins and their activated forms, which are known to be important steps in prosurvival and proliferation pathways in ovarian cancer cells. Because mTOR pathway regulates cell growth via the phosphorylation of eukaryotic initiation factor 4E-binding protein 1 (4EBP1) and ribosomal protein S6 kinase (P70S6K), we assessed the activation of the mTOR pathway by determining the phosphorylation of P70S6K and 4EBP1. CAOV-3 cells were stimulated with PAF (from 0.1 to 1000 nM) or EGF (from 0.1 to 20 ng/ml), for 24 hours. As shown in Figure 5, both PAF and EGF evoked increased levels of activated P70S6K and 4EBP1, without affecting the total amount of P70S6K and 4EBP1. AKT and ERK phosphorylation was also increased upon stimulation with PAF and EGF, respectively. However, the levels of phosphorylated β-arrestin2 were increased by PAF stimulation, but not by EGF stimulation in CAOV-3 cells.

### Combined targeting of PAFR and EGFR enhances intracellular signaling pathway inhibition

**Figure 6:**
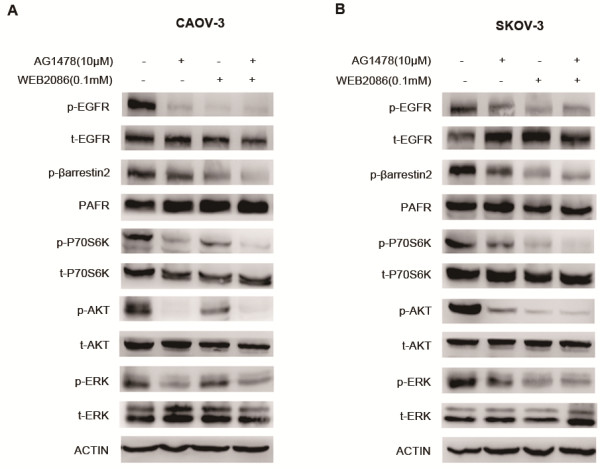
**Effects on downstream pathways by combined PAFR and EGFR targeting. (A)** CAOV-3 cell lysates were harvested 24 h after AG1478 (10 μM), WEB2086 (0.1 mM), or combination treatment. Cell lysates were subjected to immunoblotting with phospho/total -EGFR, phospho-β- arrestin2, PAFR, phospho/total-P70S6K, phospho/total-AKT, phospho/total-ERK. β-Actin was included as a loading control. **(B)** SKOV-3 cell lysates were harvested 24 h after AG1478 (10 μM), WEB2086 (0.1 mM), or combination treatment. Cell lysates were subjected to immunoblotting with the antibodies used in A.

As we have verified that both activated PAFR and EGFR can evoke the increased phosphorylation levels of P70S6K, 4EBP1, AKT and MAPK, we next determine whether the synergistic growth inhibition effects obtained by the combination of the PAFR antagonist WEB2086 and the EGFR inhibitor AG1478, were due to a more effective inhibition of intracellular signaling, and we also analysis the effect of WEB2086 and/or AG1478 treatment on the expression level of EGFR and PAFR in both cell lines. Western blot analyses were performed on proteins from CAOV-3 and SKOV-3 cells treated with 10 μM of AG1478, 0.1 mM WEB2086, or a combination with both, treatment was conducted for 24 hours. As shown in Figure 6, the combined PAFR and EGFR targeting decreased the phosphorylation levels of EGFR and β-arrestin2 compared with AG1478 or WEB2086 treatment alone, without affecting the total amount of EGFR and PAFR. β-arrestin2 has been shown to be coupled with the activated PAFR and, therefore, the phosphorylation of β-arrestin2 indicates the PAFR activation. Our results suggest that bidirectional crosstalk may occur between PAFR and EGFR. In addition, treatment with WEB2086 in combination with AG1478 resulted in a more pronounced decrease in the levels of protein phosphorylation (p-ERK, p-AKT). The combined treatment also affected mTOR signaling, as suggested by the sustained inhibition of P70S6K phosphorylation. These results suggest that combined therapy targeting PAFR and EGFR augments antitumor efficacy by inhibiting specific downstream signaling proteins.

### Co-inhibition of PAFR and EGFR significantly inhibits CAOV-3 tumor xenografts

**Figure 7:**
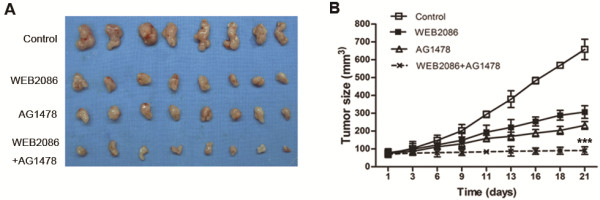
**Effects of the combined PAFR and EGFR targeting on ovarian cancer xenografts.** Athymic nude mice were injected subcutaneously into the flank with CAOV-3 cells. **(A)** Representative photographs of tumors extracted from mice treated with or without WEB2086 (5 mg/kg, i.p., every three days for 2 weeks) and AG1478 (10 mg/kg, i.p., every three days for 2 weeks). **(B)** Ovarian cancer volume was determined in mice administered WEB2086 and/or AG1478. The data represent the average (±SD). Student’s *t*-test was used to compare tumor sizes among the different treatment groups at day 21 following the start of treatment. ***, *p* < 0.001 indicates a statistically significant difference compared to the treatment with the single agent.

We finally investigated the in vivo antitumor activity of combined PAFR and EGFR targeting in nude mice bearing CAOV-3 cells that were grown subcutaneously as tumor xenografts. As shown in Figure 7, either AG1478 or WEB2086 could inhibit tumor growth compared with the control group (p < 0.001). Compared with those two treatments, the combination of AG1478 and WEB2086 led to the most significant inhibition of tumor growth (p < 0.001) at day 21 post-treatment. The combined treatment of the two drugs was well tolerated, as no obvious side effects were observed in the mice treated with AG1478 and/or WEB2086.

## Discussion

The observation that elevated levels of growth factor receptors are associated with adverse cancer outcomes has led to the development of approaches that specifically interrupt these autocrine pathways. The constitutive activation of EGFR has been reported in various cancers, including breast, prostate, and ovarian cancers [27-29]. EGFR monoclonal antibodies and EGFR tyrosine kinase inhibitors have been approved for use in cancer patients. Despite these promising preclinical results, the inhibition of EGFR, has resulted in limited antitumor effects when tested as a monotherapy in clinical settings.

The PAF/PAFR signaling axis has emerged as an important determinant of aggressive phenotypes in several malignancies [30]. PAF has been associated with early malignant transformation in BRCA1 – mutant epithelial ovarian cells [31], and melanocytic tumorigenesis has been observed in transgenic mice overexpressing PAFR [32]. The many effects of PAF in tumors, such as increased vascular permeability, the induction of neoangiogenesis, and the activation of metalloproteinases, have reinforced the concept that PAF promotes tumor metastasis [33,34]. Recent experiments have shown that the PAFR antagonist WEB2086 inhibits tumor growth in a murine melanoma model, improving overall survival when combined with chemotherapy [35].

Studies on WEB2086 have primarily been performed with leukemia cells that were induced to undergo differentiation and/or apoptosis. WEB2086 has been proven to possess the ability to abrogate PAF-mediated signals and exerts a wide anticancer activity capable of significantly decreasing proliferation in human solid tumor cells of different histogeneses and with a much higher efficacy than in normal cells. In addition, earlier experiments from our group have shown that the activation of PAFR has pleiotropic effects on tyrosine phospho-EGFR/Src/Paxillin in ovarian cancer. Therefore, we hypothesized that there would be crosstalk between the PAFR and EGFR pathways, which may be one of reasons for the resistance of cancer cells to drugs, and that the combined targeting of PAFR and EGFR would synergistically inhibit ovarian cancer progression.

In this study, we evaluated for the first time the antitumor effects of PAFR and EGFR targeting strategies in ovarian cancer cell lines using the PAFR antagonist WEB2086 and EGFR inhibitor AG1478. In our in vitro and in vivo studies, we demonstrated that EGFR and PAFR were overexpressed in ovarian cancer cell lines, which led us to speculate that simultaneously targeting PAFR and EGFR may be a more effective therapeutic strategy than targeting either signaling pathway alone. Our results show that the combined inhibition of PAFR and EGFR additively inhibited ovarian cancer progression. The decrease in viable tumor cells resulted from the induction of apoptosis, G0/G1 cell cycle arrest, and the reduction of cells in S phase.

However, the mechanisms responsible for the synergistic effects of targeting both PAFR and EGFR are not completely understood. If activated PAFR signaling acts through the EGFR signaling pathway, then EGFR targeting alone should achieve the same effect as combined targeting. The enhanced antitumor effects observed when targeting both receptors in combination suggest that EGFR-independent signaling pathways are also activated by PAF. Our results show that phosphorylation levels of P70S6K, 4EBP1, AKT, and MAPK were increased when cells were stimulated with either PAF or EGF in different doses (as shown in Figure 5). These results suggest that crosstalk exists between intracullelar signaling pathways following activation of PAFR and EGFR. With the combined treatment of WEB2086 and AG1478, the phosphorylation levels of these proteins were more reduced than with either treatment alone. Taken together, the expression of these proteins was affected by both EGFR- dependent and EGFR- independent pathways. It has been reported that after specific agonist stimulation, G protein-coupled receptors (GPCRs) use multifunctional adaptor proteins such as β-arrestins to activate many substrates in cellular pathways [36,37]. Thus β-arrestin acts as a bifunctional cellular mediator; that is, it not only terminates G protein signaling but also functions as a scaffold for transduction of the G protein signal [38]. We also observed β-arrestin2 protein was modulated by PAFR, but not EGFR, suggesting that the inhibition of the EGFR pathway alone cannot effectively suppress ovarian cancer progression.

Our previous study reported that the PAFR ligand PAF can activate phospho-EGFR and induce proliferation and invasion in ovarian cancer, and the results of the present study show that the PAFR antagonist WEB2086 can inhibit EGFR activation. It is apparent that the persistent activation of PAFR in the face of EGFR blockade still contributes to tumor growth and resistance. Identification of the proteins that are induced by PAF, in the presence or absence of EGFR inhibition, will determine the critical pathways to be targeted in combination with EGFR blockade. Further research is underway to elucidate the exact mechanism involved in this process to optimize ovarian cancer treatment regimens.

## Conclusions

Taken together, both in vitro and in vivo analyse suggest that PAFR and EGFR play an important role in the sustained growth, survival, and invasion of ovarian cancer cells. The combined usage of selective inhibitors of PAFR and EGFR, such as WEB2086 and AG1478, represents a promising strategy for the treatment of ovarian cancer. This novel combination of drugs offers a new choice for the current platinum- based regimens, but it is critical to evaluate the profile of PAFR and EGFR expression in ovarian cancer patients before the strategy is applied in the clinical setting.

## Abbreviations

PAF: Platelet-activating factor; PAFR: PAF-receptor; EGF: Epidermal growth factor; EGFR: EGF-receptor; TGF: Transforming growth factor; EMT: Epithelial mesenchymal transition; GPCR: G-protein coupled receptor (GPCR) family; PARP: Poly-ADP-ribose polymerase (PARP); 4EBP1: 4E-binding protein 1; P70S6K: Ribosomal protein S6 kinase; ERK: Extracellular-regulated protein kinase; mTOR: mammalian target of rapamycin; MAPK: Mitogen-activated protein kinases; AG1478: An EGFR-specific tyrosine kinase inhibitor; WEB2086: A specific PAFR antagonist.

## Competing interests

The authors declare that they have no competing interests.

## Authors’ contributions

YY performed the experiments and drafted the manuscript. XYZ and SSH participated in the design of this study. MXZ and QQC participated in the experiments. WJ and CJX contributed to the design of this study, final data analysis and edited the manuscript. All authors read and approved the final manuscript.
